# Comparative Transcriptomic Analysis of Male and Female Gonads in *Hemibagrus guttatus* (Lacepède)

**DOI:** 10.3390/ani15243541

**Published:** 2025-12-09

**Authors:** Wenyin Luo, Shaojun Huang, Guanglve Li, Dan Hu, Jiemei Chen, Huiqin Li, Hemin Yu, Yanyun Chen, Jiajie Zhu, Qiaomu Hu

**Affiliations:** 1 Guangxi Key Laboratory of Polysaccharide Materials and Modification, Guangxi Minzu University, Nanning 530004, China; luowenyin7235@163.com (W.L.);; 2 State Key Laboratory of Mariculture Biobreeding and Sustainable Goods, Yellow Sea Fisheries Research Institute, Chinese Academy of Fishery Sciences, Qingdao 266071, China

**Keywords:** *Hemibagrus guttatus*, transcriptomics, gonadal differentiation and development, gene expression

## Abstract

*Hemibagrus guttatus* is a commercially valuable freshwater fish in the Pearl River Basin, renowned as the “King of Freshwater Fish.” Due to habitat degradation and overfishing, its wild population has declined sharply, leading to its listing as a National Key Protected Wild Animal of Class II in China. Artificial breeding is therefore crucial for conservation, yet progress is hindered by the lack of clear sexual dimorphism and poor understanding of its sex differentiation mechanism. In this study, we performed high-throughput RNA sequencing (RNA-seq) to compare gonadal transcriptomes of male and female *H. guttatus*. A total of 3245 differentially expressed genes (DEGs) were identified, including 3122 male-biased and 123 female-biased DEGs, which clustered into three distinct expression patterns. Enrichment analysis revealed that genes associated with the TGF-β (Transforming Growth Factor-beta) and GnRH (Gonadotropin-Releasing Hormone) signaling pathways were significantly enriched in the female gonads, suggesting their potential roles in gonadal differentiation. From the DEG set, we further highlighted five genes with pronounced sex-biased expression: *rbm46* (RNA Binding Motif Protein 46) exhibited gonad-specific expression, whereas *myc* (v-myc avian myelocytomatosis viral oncogene homolog), *angptl4* (Angiopoietin-Like 4), *sox9* (SRY-Related HMG-Box Gene 9), and *fzd2* (Frizzled Class Receptor 2) showed marked expression differences between male and female gonads. These findings provide insights into the molecular mechanisms underlying sex differentiation in *H. guttatus*, offer potential molecular markers for sex identification, and establish a scientific basis for germplasm conservation and the optimization of breeding techniques.

## Simple Summary

*Hemibagrus guttatus*, recognized as one of China’s Pearl River ‘Four Famous Fishes’, faces challenges in artificial breeding and monosexual cultivation due to the difficulty in identifying sexual phenotypes and the limited understanding of sex determination and differentiation mechanisms. To address this, we employed high-throughput RNA sequencing (RNA-seq) to analyze and compare the gonadal transcriptomes of male and female spotted *H. guttatus*, aiming to identify key genes and signaling pathways involved in sex differentiation. Our findings not only reveal the molecular basis of sex differentiation in this species but also provide potential for developing specific molecular markers and constructing regulatory networks. These advances establish a scientific foundation for conserving germplasm resources and implementing artificial breeding practices.

## 1. Introduction

*Hemibagrus guttatus* (Lacepède, 1803) was a common freshwater fish in China’s Yangtze River Basin during the 1990s [1]. It is known as the ‘King of Freshwater Fish’ due to its habitat in rocky caves [2], preference for clean water, mud-free flavor, and abundant gelatinous tissue at the bone–meat interface [3,4]. Due to habitat destruction and overfishing, which have led to a significant decline in the wild population of *H. guttatus*, it was classified as a Class II Key Protected Wild Animal in China in 2021. Despite its high commercial aquaculture potential, *H. guttatus* artificial breeding techniques remain underdeveloped, as practices primarily depend on wild broodstock for induced spawning. This severely limits population recovery and sustainable utilization. Thus, elucidating the sex determination mechanism and identifying reliable sex-related markers in *H. guttatus* are crucial for advancing its artificial breeding, monosex culture, and population conservation.

In teleost fish, sex determination and differentiation mechanisms exhibit high plasticity, which complicates their study, as they are regulated not only by genetic factors (such as sex chromosomes or autosomal genes) but also are susceptible to environmental influences such as temperature, light exposure, and population structure [5]. Fish within the family *Bagridae* (order *Siluriformes*) predominantly exhibit XX/XY male heterogametic sex determination systems. The core sex determination genes (*amhr2*/*amhr2y*, *amh*) are concentrated within the TGF-β pathway and transcription factor families (*dmrt1*, *sox9*) [6,7]. As a core group within Siluriformes, known sex-determination mechanisms and identified sex-associated markers in Bagridae provide a basis for developing monosex aquaculture. Research has focused on the sex regulation mechanisms of economically important species [8,9], offering a solid reference for elucidating the molecular mechanisms of sex differentiation in *H. guttatus*.

Notably, *H. guttatus* exhibits indistinct secondary sexual characteristics, making visual sex identification challenging and complicating parental selection and artificial breeding. The recent completion of male and female chromosome-level genome assemblies of both sexes for this species by the Pearl River Fisheries Research Institute [10] has provided the data foundation for elucidating its sex regulation mechanisms at the genomic and transcriptomic levels. Therefore, the lack of distinct morphological markers in *H. guttatus* not only hinders practical breeding but also underscores a critical knowledge gap: the molecular drivers of its sex differentiation remain uncharacterized. This gap makes transcriptomic analysis particularly suitable, as it directly addresses the problem by enabling the systematic comparison of mRNA expression profiles between sexes to identify key regulatory genes and pathways without relying on physical traits. Against this backdrop, the present study compares transcriptomic profiles of male and female gonads to identify sex-biased genes and regulatory pathways, with the goal of clarifying the molecular mechanisms underlying sex determination in *H. guttatus*. These findings will support the development of artificial breeding technologies and contribute to germplasm conservation for this species.

## 2. Materials and Methods

### 2.1. Experimental Animals and Tissue Collection

*H. guttatus* (average size 780 ± 12 g) at 1.5 years of age were collected from a suburban fish breeding farm (Nanning, Guangxi Autonomous Region, China), and the experiment was approved by the Experimental Animal Care, Ethics and Safety Committee (No. YSFRI-2025017).

Before the experiment began, the fish were reared in the laboratory for two weeks in a controlled recirculating flow-through with a tank capacity of 500 L, a photoperiod of 12 h light: 12 h dark, water system with the water temperature set at 24 °C and a pH range of 6.8–7.2, DO(dissolved oxygen) of 6.3–8.5 mg/L, NO_2_-N(nitrite) of 0.01–0.03 mg/L, and NH_4_-N(ammonium nitrogen) of 0.02–0.05 mg/L. To ensure three biological replicates of males and females, we selected 20 healthy, uninjured individuals from the farm. Post-dissection, we found a significant sex bias among the 20 individuals: 16 were female and only 4 were male, with one male having immature gonads. Only anatomized samples with mature gonads were used, excluding those that were immature or overmature. The criteria for judging gonadal maturity were as follows: mature males had paired, milky white to pale yellow testes with compact texture and distinct lobular structures, and a small amount of milky semen could be extruded by light abdominal pressure; mature females had yellowish-orange ovaries with soft texture and visible mature follicles (follicle diameter > 0.8 mm) under stereomicroscopy, without atresia or overripening.

MS-222 (Sinopharm Chemical Reagent Co., Ltd., Shanghai, China) was prepared at a concentration of 110 mg/L to anesthetize the experimental fish. The tissues of gonad, liver, brain, heart, kidney, intestine, stomach, gill filament, and seminal vesicles were collected from three female and three male *H. guttatus* and then immediately frozen in liquid nitrogen. The samples were stored in an ultra-low temperature freezer at −80 °C to ensure sample integrity and facilitate the extraction of total RNA. For subsequent functional validation, the gonadal tissue samples were divided into two halves: one half underwent transcriptomic sequencing, while the other half was stored as a backup for future qPCR experiments.

### 2.2. RNA Isolation, Library Construction and Sequencing

The total RNA was extracted using the RNAsimple Total RNA Kit (TIANGEN BIOTECH (BEIJING) Co., Ltd., Beijing, China) following the manufacturer’s instructions. RNA integrity was assessed by 1% agarose gel electrophoresis, and RNA concentration was measured using an Agilent 2100 Bioanalyzer (Agilent Technologies Inc., Santa Clara, CA, USA). RNA-seq libraries were constructed from three independent biological replicates per sex (6 libraries total, no sample pooling during RNA extraction or library construction).

RNA-seq libraries were prepared and sequenced at Zhenyue Biotechnology Laboratory (Wuhan, China). cDNA libraries were prepared using the cDNA Library Construction Kit (TaKaRa Biotechnology (Dalian) Co., Ltd., Dalian, China), with an initial total RNA amount ranging from 2.14 to 3.25 µg. PCR amplification was performed for 15 cycles, and the final concentration of amplified libraries was 19.8–21.4 ng/µL in a 30 µL volume, corresponding to a total library yield of 594–642 ng, with an average fragment length (insert size) of 270 bp. The final amplified libraries were quantified and adjusted using the NGS Library Quantification Kit (Kapa Biosystems, Inc., Wilmington, MA, USA) and sequenced on the BGISEQ-500 instrument (BGI Genomics Co., Ltd., Shenzhen, China) using a 150 bp paired-end (PE) sequencing strategy. Based on the BGISEQ-500 platform’s 150 bp PE sequencing mode (platform-recommended for transcriptome sequencing), clean data volume (5.95–8.39 Gb per sample), and a typical 5–10% sequencing data filtering rate, the raw read count per sample was 20.9–31.1 million reads. The average sequencing coverage depth per sample was 4.77×–5.60×, based on the *H. guttatus* reference genome (749.1 Mb) and clean data volume.

### 2.3. Assembly and Sequence Annotation

Raw reads were quality-filtered with fastp (v0.23.2) by removing adapters, trimming Poly-G tails, correcting overlapping bases of paired-end reads, and excluding low-quality reads (quality score < 20; length < 30 bp).

The high-quality clean reads were mapped to the *H. guttatus* reference genome (NCBI Assembly ID: GCA_033459395.1; Version: 1.0) using HISAT2 (v2.1.0) to evaluate the mapping results, including the genomic distribution of reads, gene body coverage, sequencing saturation, and alignment statistics. Reads with a mapping quality score below 10, improperly aligned reads, and reads mapping to multiple regions of the genome were filtered out.

The feature Counts tool from the subread software (v2.0.1) was used to quantify gene expression levels for each sample, generating the raw expression matrices and FPKM (fragments per kilobase of transcript per million) for all samples. After obtaining the FPKM values for all genes across samples, we first performed sample correlation analysis to ensure more reliable results in subsequent differential gene analysis. Using the R package pheatmap (v1.0.12), we calculated Pearson correlation coefficients based on the gene expression matrix and plotted a sample correlation heatmap.

Since gene annotations are not universal across databases, integrating multi-database annotations enables more comprehensive characterization of their functions and properties. Annotating genes with Diamond (v2.0.14.152) against the Non-Redundant (NR) Database (https://ftp.ncbi.nlm.nih.gov/blast/db/FASTA/, accessed on 10 January 2024) and Swiss-Prot (https://ftp.ncbi.nlm.nih.gov/blast/db/FASTA/, accessed on 10 January 2024) reveals the species-specific homolog distribution of sequenced genes fragments in known protein databases; annotating against the AnimalTFDB Database (https://guolab.wchscu.cn/AnimalTFDB4/#/, accessed on 10 January 2024) provides transcription factor family annotations and their quantitative distributions; annotating to the EggNOG Database (http://eggnog5.embl.de/#/app/home, accessed on 10 January 2024) clarifies the distribution of isoforms across functional categories under KOG classification; annotating to the KEGG Database (https://www.genome.jp/kegg/, accessed on 10 January 2024) yields gene annotations and their quantitative distributions across metabolic pathways and functional categories; annotating to the GO Database (https://www.geneontology.org/, accessed on 10 January 2024) obtains gene annotations and their quantitative distributions across the three ontologies: biological process, cellular component, and molecular function. Cross-referencing the Diamond-derived annotations from the aforementioned GO, KEGG, NR, EggNOG, and Swiss-Prot Databases using the R package VennDiagram (v1.7.3) identifies the distribution of genes with overlapping annotations, where higher overlap indicates greater reliability of gene annotations.

### 2.4. Methodology for Identification of Differentially Expressed Genes

Differential expression analysis was performed using the R package DESeq2 (v1.24.0), with raw gene count matrices generated by featureCounts (row names: NCBI Gene IDs; column names: samples). All downstream analyses, including the assessment of sample relationships, were conducted based on these raw counts and the variance-stabilizing transformation (VST) data generated by DESeq2, ensuring methodological consistency. First, low-expression genes (row means ≤ 1) were filtered out to reduce background noise and improve the reliability of subsequent differential analysis. The “sex” grouping factor was then converted to R’s factor type to construct a colData frame, which included 3 female and 3 male biological replicates.

Prior to formal differential analysis, we evaluated potential batch effects using sample correlation analysis and hierarchical clustering based on the VST-normalized count matrix. All samples were subjected to library construction and sequencing in a single batch, avoiding the introduction of technical batch variation. The sample correlation heatmap further confirmed that samples clustered primarily by sex (females: C-1–C-3; males: X-1–X-3) with no batch-specific grouping, indicating no significant batch effects and obviating the need for additional batch correction.

We next created a DESeqDataSet object with the design formula “~sex” and used the DESeq() function (parameters: fitType = “mean”, minReplicatesForReplace = 7, parallel = FALSE) for analysis. This function internally completes key steps including size factor-based normalization, dispersion estimation, negative binomial generalized linear model fitting, and Wald test for differential expression. *p*-values were adjusted using the Benjamini–Hochberg procedure to control the false discovery rate (FDR). Statistically significant DEGs were defined with the thresholds |log_2_(FoldChange)| ≥ 1.0 and padj ≤ 0.05; these DEGs were further subdivided into male-highly expressed and female-highly expressed subsets.

DEGs often share common functions or participate in the same metabolic and signaling pathways. We employed agglomerative hierarchical clustering [11] for the DEGs set, with row normalization performed on the expression data to group genes with similar expression patterns together. Specifically, the H-cluster method was used to partition the DEGs set into multiple clusters. This method calculates distance matrices between genes and samples using the Pearson correlation coefficient, then identifies genes with similar expression patterns from the analysis results.

### 2.5. Enrichment Analyses

The differential gene sets were analyzed for GO and KEGG enrichment using the hypergeometric distribution test, with a FDR ≤ 0.05 indicating that the differential genes were significantly enriched in the GO terms or KEGG signaling pathways associated with them. The clusterProfiler software (v4.8.1) was used to perform multiple-testing correction with the Benjamini–Hochberg method, while OrgDb (v 3.18.0) was employed to map differential genes to annotated GO terms and KEGG pathways.

GSEA (Gene Set Enrichment Analysis) was conducted using an empirical phenotype permutation test, also with the clusterProfile software (v4.8.1). The adaptive multilevel splitting Monte Carlo approach algorithm from the fgsea package (v1.28.0) was used to calculate *p*-values, which were subsequently adjusted using the Benjamini–Hochberg method.

### 2.6. Quantitative Real-Time PCR

To validate the results of the differential expression analysis, qPCR (quantitative real-time PCR) was performed to screen potentially sex-specific DEGs. Tissues including liver, brain, heart, kidney, intestine, stomach, gill filament, ovary (females only) and testis (males only) were collected from each individual. Total RNA was extracted from the above tissues. Genomic DNA was removed, and reverse transcription was conducted using the PrimeScript™ FAST RT reagent Kit (TaKaRa Biotechnology (Dalian) Co., Ltd., Dalian, China) according to the manufacturer’s instructions. qPCR was performed using the FQD-96C instrument (Hangzhou Bioer Technology Co., Ltd., Hangzhou, China) and THUNDERBIRD™ Next SYBR^®^ qPCR Mix (TOYOBO Co., Ltd., Shanghai, China). Three biological replicates were included for each reaction. EF-1α was used as the reference gene, and relative expression levels were calculated using the 2−ΔΔCt method. Prior to statistical analysis, the normality of the 2−ΔΔCt data distribution was assessed using the Shapiro–Wilk test, and the homogeneity of variances was evaluated using Levene’s test. As the data met the assumptions of normality and homoscedasticity, differential expression was statistically confirmed by one-way analysis of variance (ANOVA) followed by Tukey’s post hoc test for multiple comparisons. The primer sequences used in this study are provided in Table 1.

## 3. Results

### 3.1. Assessment of Sequencing Quality

Male and female samples of *H. guttatus* were subjected to RNA sequencing on a BGISEQ-500 instrument. Reads from different samples were distinguished using multiple identifier (MID) tags. A total of 294.8 million pairs of 150 bp high-quality reads were generated on a single channel for *H. guttatus*. Across six samples, 44.03 Gb of clean reads were obtained, with each sample yielding more than 5.9 Gb of clean reads. The percentage of bases with Q30 (sequencing error rate < 0.1%) was 89.5% or more (Table 2). The clean reads showed high alignment rates (90.10–95.91%) to the reference genome (Table 3).

**Figure 1:**
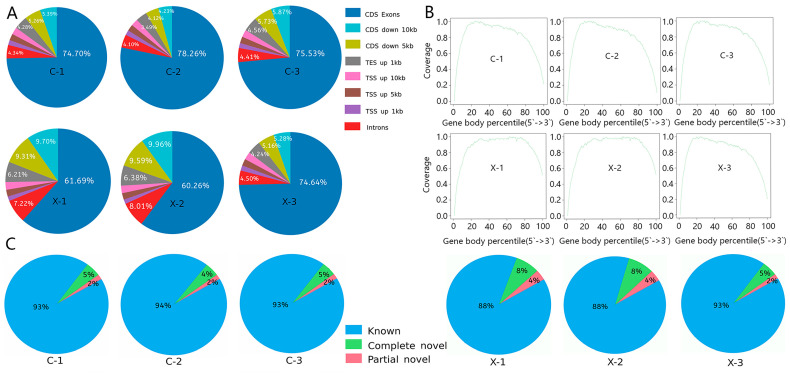
Alignment of sequencing reads from male and female samples to the reference genome: (**A**) Components and ratios of sequencing reads for male and female samples; (**B**) Distribution of sequenced reads across gene bodies in male and female samples; (**C**) Statistical analysis of annotated splicing events in male and female samples.

Genomic distribution analysis indicated consistent mapping patterns across samples, with over 60% of reads aligning to coding exons (Figure 1A), confirming the enrichment of mature mRNA. Analysis of splicing events (Figure 1C) splicing types constituted >88% of all events, which is consistent with the use of a well-annotated reference genome.

Having confirmed the high quality and reliability of our sequencing data, we next sought to characterize the global gene expression profiles and investigate the overall transcriptional similarities and differences between male and female gonads.

### 3.2. Quantitative Analysis and Annotation

**Figure 2:**
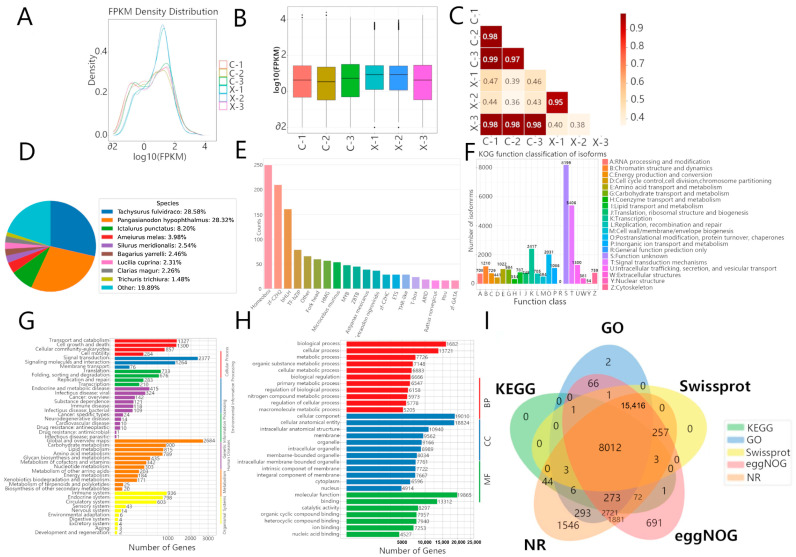
Distribution of gene expression levels among different samples based on the samples’ FPKM matrices: (**A**) Plotting of density distribution curves based on FPKM from each sample; (**B**) Plotting of boxplots based on FPKM from each sample; (**C**) Heatmap of correlations among samples, in which the darker the color and the closer the value to 1, the more similar the gene expression patterns are among the samples. Annotation of genes to multiple biological databases: (**D**) Query of all genes against the Non-Redundant Protein Sequence Database (NR) and Swiss-Prot Protein Knowledgebase (Swiss-Prot) databases; (**E**) Matching of all genes to the Transcription Factor (TF) Database; (**F**) Annotation of all genes to the EggNog database; (**G**) Annotation of all genes to the Kyoto Encyclopedia of Genes and Genomes (KEGG) database; (**H**) Mapping of all genes to the three ontologies of the Gene Ontology (GO) database; (**I**) All genes with overlapping annotations via cross-referencing to the GO, KEGG, NR, EggNOG, and Swiss-Prot databases.

Gene expression was quantified for each sample (Table 4). To assess data reproducibility and inter-sample relationships, we analyzed the distribution and correlation of expression levels (log_10_(FPKM)). Density plots and boxplots revealed variation in expression distributions among samples (Figure 2A,B). Sample correlation analysis clustered the replicates into two primary groups: one containing samples X-1 and X-2 (r = 0.95), and another containing samples C-1, C-2, C-3, and the male sample X-3 (r ≥ 0.97) (Figure 2C).

We noted that one male sample (X-3) clustered with the females. To address whether this resulted from technical artifacts, we examined two key factors. First, all libraries were prepared and sequenced in a single batch, eliminating batch effects as a cause (see Section 2.4 Methodology for Identification of Differentially Expressed Genes). Second, the sex of X-3 was unequivocally confirmed by direct anatomical inspection of mature testes at dissection, ruling out sex misidentification. Therefore, the expression profile of X-3 likely reflects underlying biological variation, such as individual differences in gonadal maturation or genetic background, which is not uncommon in outbred aquaculture populations. Crucially, the high correlation within the core male (X-1, X-2) and female (C-1, C-2, C-3) groups provides a robust foundation for identifying sex-biased expression patterns, and the statistical model (DESeq2) used for differential analysis accounts for variance within groups.

Since genes are not annotated in all databases, integrating multiple database annotations can provide a more comprehensive view of the functionality of genes and features. Using diamond, 34,404 genes were searched against multiple databases such as NR, Swiss-Prot, GO, KOG, and KEGG with an E-value of 1 × 10^−5^.

As shown in Figure 2D–I, 30,530 genes (88.74%) were found to be annotated in NR, and 23,696 genes (69.88%) (Figure 2D) in Swiss-Prot. The results showed the highest homology with catfish species (*Tachysurus fulvidraco*, *Pangasianodon hypophthalmus*), with the remaining 19.89% of genes showing no close homology to these species. In TrEMBL, 29,242 genes (85.00%) were annotated, and 1740 genes were annotated in the TF database and assigned to 20 gene families, with the top three being Homeobox (250 genes), zf-C2H2 (210 genes), and bHLH (161 genes) (Figure 2E).

In EggNOG, 29,395 genes (85.44%) were annotated and assigned to 21 subcategories, with the top three being Function unknown (8199 genes), Signal transduction mechanism (5406 genes), and Transcription (2417 genes), as well as Posttranslational modification, protein turnover, and chaperones (2031 genes) (Figure 2F). Additionally, 19,968 genes were assigned to KO pathway classes in Cellular Processes, Environmental Information Processing, Human Diseases, Metabolism, and Organismal Systems, and Lipid metabolism (815 genes) (Figure 2G). In the GO database, 13,858 GO terms were annotated, covering Molecular Function, Biological Process, and Cellular Component (Figure 2H).

Among genes annotated by multiple bioinformatic databases, 8012 genes had similar or related biological functions. EggNOG provided classification and functional annotation of orthologous genes, with 691 independently annotated genes; NR, which contains more computational predictions, had 1546 independently annotated genes (Figure 2I).

### 3.3. Identification of Differentially Expressed Genes

**Figure 3:**
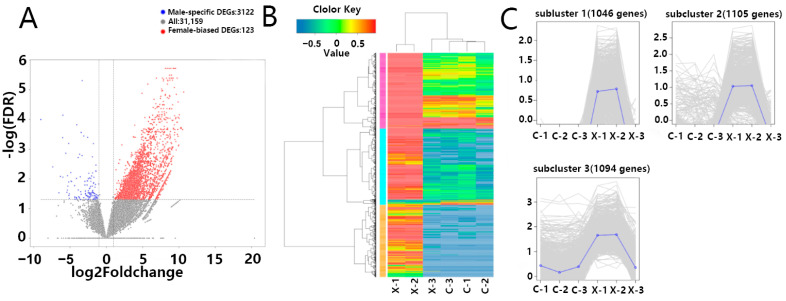
Differentially Expressed Gene Analysis and Identification: (**A**) Significant differential analysis of quantified genes between male and female samples, generating a volcano plot where red dots represent up-regulated genes and green dots represent down-regulated genes; (**B**) Cluster analysis of DEGs yielding a clustering heatmap, with red indicating high gene expression and green indicating low gene expression; (**C**) The set of DEGs was divided into three clusters using the Hierarchical cluster (H-cluster) method, with genes in the same cluster showing similar expression patterns under different treatment conditions.

Using DESeq2 (v1.24.0), a total of 3245 DEGs were identified, comprising 3122 male-biased DEGs and 123 female-biased DEGs (Figure 3A). To visualize gene expression patterns, we first extracted the normalized expression matrix obtained from DESeq2, and then generated a hierarchical clustering heatmap of the DEGs set using pheatmap (Figure 3B). C-1, C-2, C-3, and X-3 cluster together (predominantly blue, indicating low expression), while X-1 and X-2 form a separate branch (predominantly red, indicating high expression). Cross-branch comparison identifies shared highly expressed gene clusters; most DEGs are highly expressed in X-1 and X-2, with only a few clusters showing female-biased high expression in C-1, C-2, and C-3.

Subsequently, H-clustering grouped the differential gene set into clusters with similar expression patterns across samples. The three subclusters in Figure 3C correspond to the major gene clusters in Figure 3B: Subcluster_1 (1046 genes), where C-1, C-2, C-3, and X-3 show mean-centered expression (≈0) while X-1 and X-2 exhibit high expression; Subcluster_2 (1105 genes), showing a similar pattern to Subcluster_1 but with more dispersed expression profiles; and Subcluster_3 (1094 genes), expressed in both sexes but at significantly higher levels in X-1 and X-2.

The identification of thousands of DEGs underscored the profound transcriptomic divergence between male and female gonads. We then focused on elucidating the biological functions and signaling pathways these DEGs are involved in, to uncover the molecular mechanisms underlying gonadal differentiation.

### 3.4. Enrichment Analysis of DEGs

**Figure 4:**
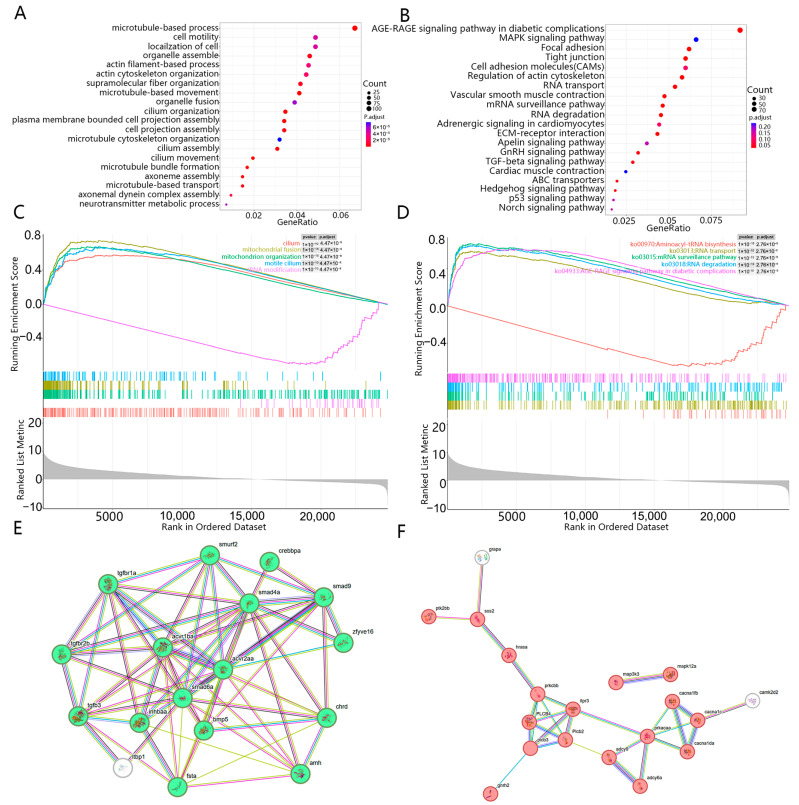
Enrichment analysis of differential gene sets to screen out key pathways and biological processes: (**A**) Gene Ontology (GO) enrichment analysis of DEGs in the female and male gonads of *H. guttatus*; (**B**) The most enriched KEGG pathway from DEGs in the gonads of female and male *H. guttatus*; (**C**) GSEA-GO enrichment analysis of DEGs in the female and male gonads of *H. guttatus*; (**D**) GSEA-KEGG enrichment analysis of DEGs in the female and male gonads of *H. guttatus*; (**E**) A regulatory network composed of DEGs annotated to the TGF-beta signaling pathway. spherical shapes represent proteins, connecting lines represent the interaction relationships between two proteins, and green spherical proteins are the core proteins in the STRING regulatory network; (**F**) A regulatory network composed of DEGs annotated to the GnRH signaling pathway. Spherical shapes represent proteins, lines represent the interaction relationships between two proteins, and spherical proteins in red are the core proteins in the STRING regulatory network.

Functional annotation of 3245 DEGs yielded 3508 significant GO terms (padj < 0.05). These were predominantly in the cellular component category (2483 terms, 70.8%), followed by molecular function (654 terms, 18.5%) and biological process (371 terms, 9.8%) (Figure 4A). Among the most significantly enriched biological processes were terms directly related to male reproduction, including “spermatogenesis” (GO:0007283), “male gamete generation” (GO:0048232), and “spermatid differentiation” (GO:0048515). Other prevalent enriched terms across the ontologies included “microtubule-based processes” (GO:0007017), “cilium” (GO:0005929), and “actin-binding” (GO:0051015). The robust enrichment of spermatogenesis-related terms suggests that the male gonads were undergoing active sperm production at the sampled stage.

KEGG pathway analysis identified several significantly enriched pathways, including “Focal adhesion”, “Tight junction”, “Regulation of actin cytoskeleton”, and “Vascular smooth muscle contraction” (Figure 4B). Notably, the “TGF-beta signaling pathway” and the “GnRH signaling pathway” were also significantly enriched, with 26 and 25 DEGs annotated to them, respectively. Protein–protein interaction networks constructed for genes in these pathways revealed cohesive clusters (Figure 4E,F). The TGF-beta cluster included key members of the signaling cascade (e.g., *acvr2aa*, *smad6a*, *bmp5*, *inhbaa*). Given the established role of TGF-β superfamily genes in fish sex differentiation, this enrichment may indicate a role for this pathway in male gonadal development of *H. guttatus*. Similarly, the GnRH signaling pathway cluster contained genes involved in intracellular signal transduction (e.g., *plcb2*, *plcb3*, *plcb4*). This, together with the pathway’s known function in the reproductive axis, suggests its potential involvement in gonadal regulation.

Gene Set Enrichment Analysis (GSEA) provided a complementary perspective by assessing coordinated expression changes across predefined gene sets. GSEA highlighted significant enrichment of gene sets related to mitochondrial organization (e.g., mitochondrial fusion, GO:0008053) and ribosomal/translational processes (e.g., Aminoacyl-tRNA biosynthesis, ko00970), which were not prominently identified by the conventional hypergeometric enrichment analysis (Figure 4C,D).

Following differential analysis, we identified 3122 male-biased DEGs and 123 female-biased DEGs. Based on the DEGs clustered in Cluster 3 and enrichment analysis results, we filtered out numerous gonadal housekeeping genes annotated to GTP cycling and metabolic changes. Subsequently, by examining the DEGs clustered in Cluster 1 and Cluster 2, as well as the strongly correlated gene classification results in the clustering heatmap, we selected *myc* (male-biased), *rbm46* (female-biased), *angptl4* (female-biased), *sox9* (male-biased), and *fzd2* (male-biased). These genes are sex-differentially expressed and belong to the same cluster, and they lack extensive annotation in fundamental cellular activity pathways. Thus, they are preliminarily identified as potential sex-specific DEGs strongly associated with the gonad. We used qPCR to verify the RNA-seq results and examine the tissue expression patterns of these candidate genes in male and female *H. guttatus*.

### 3.5. Validation of Sex Differentiation Genes by qRT-PCR

**Figure 5:**
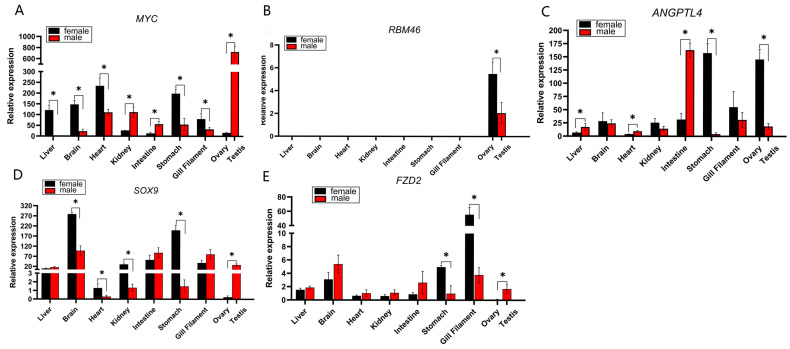
Relative expression of sex-related genes in various tissues of *H. guttatus*: (**A**) Relative expression of the *myc* gene in various tissues of *H. guttatus*; (**B**) Relative expression of the *rbm46* gene in various tissues of *H. guttatus*; (**C**) Relative expression of the *angptl4* gene in various tissues of *H. guttatus*; (**D**) Relative expression of the *sox9* gene in various tissues of *H. guttatus*; (**E**) Relative expression of the *fzd2* gene in various tissues of *H. guttatus*. Asterisks indicate significant differences in gene expression (*p* < 0.05).

The DEGs identified by RNA-Seq were verified by qPCR. The amplification curves showed S-shaped curves, the melting curves showed single peaks, and all the genes tested were single products. Liver, brain, heart, kidney, intestine, stomach, gill filament, ovary and testis tissues were taken from the *H. guttatus*, and the qPCR results of the five genes are shown in Figure 5.

The expression profile of *myc* revealed a complex pattern of sexual dimorphism. While it was predominantly expressed at higher levels in females across several somatic tissues (e.g., liver, brain, heart), its expression was strikingly biased towards males in the gonads, with testicular levels being 45-fold higher than in the ovary (Figure 5A). This stark contrast suggests a distinct, gonad-specific regulatory mechanism for *myc* that overrides its expression trend in somatic tissues.

qPCR results confirmed the strict gonad-specific expression of *rbm46*, as no expression was detected in any of the examined non-gonadal tissues (Figure 5B). Furthermore, the expression level of *rbm46* in the ovary was significantly higher than that in the testis (*p* < 0.05). This female-biased expression pattern in the gonads aligns with its established role in regulating meiosis and maintaining oocyte development in other teleost fish, highlighting the gene’s potential critical function in female gametogenesis in *H. guttatus*.

Expression of *angptl4* was detected in multiple tissues, with the highest levels in the ovary and significant levels in the male intestine and female stomach (Figure 5C). Critically, ovarian expression significantly exceeded that in the testis (*p* < 0.05). Given its central role in lipid metabolism and energy homeostasis, the strong ovarian expression of *angptl4* suggests a crucial function in mobilizing lipid resources to support vitellogenesis and follicular development.

The expression pattern of *sox9* is complex, exhibiting a marked female bias across multiple tissues (Figure 5D). However, in the gonads, this trend was completely reversed, with testicular expression levels being 140-fold higher than in the ovary (*p* < 0.05). This dramatic reversal underscores that *sox9* is subject to a potent, testis-specific regulatory program, which is consistent with its well-established, conserved function in driving testicular differentiation and spermatogenesis in vertebrates.

While *fzd2* was ubiquitously expressed at low levels, it exhibited significant enrichment in the testis compared to the ovary (Figure 5E). This testis-specific enrichment of a key Wnt pathway receptor implies a functional requirement in male reproductive development, potentially in processes such as spermatogonia stem cell maintenance or testicular differentiation.

## 4. Discussion

*H. guttatus* is currently listed as a National Key Protected Wild Animal of Class II in China, but studies on its gonadal differentiation and development remain limited. Our transcriptomic analysis revealed a notable case of individual variation: the phenotypic male sample X-3 consistently clustered with female samples in expression profiles (Figure 2C and Figure 3B). Technical confounders such as batch effects or sex misidentification were ruled out (see Section 2.4 Methodology for Identification of Differentially Expressed Genes). This pattern most parsimoniously reflects substantial biological variation among male individuals in this species, which could arise from factors like gonadal maturation stage or genetic background. While sex reversal is a phenomenon documented in some teleost [12], our transcriptomic data alone cannot support this specific hypothesis over other explanations for individual differences. Therefore, we interpret this finding primarily as evidence of natural transcriptional heterogeneity within the male sex of *H. guttatus*.

A key limitation of this study is the modest sample size (three replicates per sex). Although sufficient for identifying robust DEGs, it may affect the detection of subtle expression differences and limits generalizability, as highlighted by the variable sample X-3. Future studies with larger cohorts across different seasons and developmental stages are essential to validate our candidate genes and fully elucidate the transcriptional basis of gonadal development in *H. guttatus*.

### 4.1. Identification of the DEGs

Based on our transcriptomic data, we identified key sex-related DEGs (*dmrt1*, *nr5a1*, *sox3*, *cyp19a1*) whose species-specific expression patterns directly reflect the molecular characteristics of gonadal differentiation in *H. guttatus*. These genes were screened from 3245 DEGs identified by RNA-seq, with expression trends that either align with conserved vertebrate sex regulation or exhibit unique features of this species, providing a foundation for deciphering its sex determination mechanism.

The expression of *dmrt1*, a conserved regulator of male gonadal development in vertebrates [13,14,15,16,17], showed a distinct pattern in *H. guttatus*: it was specifically upregulated in female gonads. While this contrasts with its canonical pro-male role, a similar female-associated or context-dependent expression of *dmrt1* has been documented in other teleost, such as in *Acipenser baeri* (co-expressed with female-biased genes) and *Anguilla japonica* (expressed in early oogonia) [18,19]. Therefore, in *H. guttatus*, the strong female-biased expression of *dmrt1* may indicate a divergent, species-specific function within the ovarian regulatory network. Its precise role warrants further investigation.

The nuclear receptor *nr5a1* (SF-1), a key regulator of steroidogenic gene expression [20,21], was also upregulated in female *H. guttatus* gonads. This female-biased expression, alongside its co-upregulation with *dmrt1*, presents a distinctive profile in this species. In our data, *nr5a1* was co-expressed with *cyp19a1a*, a key estrogen synthase gene that was also female-biased. This is consistent with the conserved role of *nr5a1* in promoting estrogen synthesis pathways in teleost [22,23]. The female-biased co-expression of *dmrt1* and *nr5a1* suggests they may be part of a shared regulatory program in *H. guttatus* ovaries. Their expression could potentially be influenced by endocrine signals, though this requires direct experimental verification [24,25,26,27].

Consistent with the patterns shown in Figure 3A, *sox3* showed moderate male-biased expression in *H. guttatus*. This aligns with its reported role in testis development in specific teleosts such as in the genus *Acanthopagrus* [28]. Conversely, *cyp19a1* (aromatase) exhibited clear female-biased expression. This pattern is congruent with its conserved function, exemplified by its ovarian-specific expression in species like *Trichogaster trichopterus* and its fundamental role in estrogen synthesis and ovarian maintenance [29,30,31].

### 4.2. Validation of Sex Differentiation Genes by RT-qPCR

To confirm the reliability of the DEGs identified in Section 4.1 and clarify their tissue-specific expression patterns, we selected 5 core candidate genes (*myc*, *rbm46*, *angptl4*, *sox9*, *fzd2*) for qPCR validation across multiple tissues. These genes were chosen for their significant sex bias in transcriptomic data and coverage of different functional categories, ensuring they could comprehensively reflect the sex-specific expression landscape of *H. guttatus.*

qPCR validation confirmed *myc*’s ubiquitous somatic expression but striking gonadal sexual dimorphism—testicular levels were 45-fold higher than in ovaries (Figure 5A), aligning with its transcriptomic male bias. As a conserved metabolic transcription factor [32], *myc* drives aerobic glycolysis and lipid synthesis [33]. Its marked enrichment in testes suggests a role in supporting the heightened metabolic and biosynthetic demands of active spermatogenesis in *H. guttatus*.

*rbm46* showed strict gonad-specific expression in *H. guttatus* (no somatic expression detected, Figure 5B), with significantly higher ovarian abundance (*p* < 0.05)—a pattern consistent with its conserved role in teleost gametogenesis [34]. In zebrafish, *rbm46* mutants exhibit female-to-male sex reversal and meiotic arrest, and the gene regulates germ cell mRNA stability; genetic restoration partially rescues meiotic defects [34], highlighting conserved functions in fish meiosis. For *H. guttatus*, this gonad restriction (even at low expression levels) underscores a specialized role in gametogenesis: ovarian enrichment suggests it maintains oocyte meiotic progression, while low testicular expression may support spermatocyte maturation—aligning with *rbm46*’s established status as a key germ cell regulator [35].

*angptl4* exhibited seven-fold higher expression in *H. guttatus* ovaries than testes (Figure 5C), consistent with its transcriptomic female bias and core role in lipid metabolism. As a regulator of lipoprotein lipase (lpl) [36], *angptl4* modulates lipid mobilization—essential for *H. guttatus* ovarian vitellogenesis (mature follicle diameter > 0.8 mm, Section 2.1). In teleost, lpl promotes gonadal tissue activity [37,38,39], and *angptl4* indirectly upregulates mmp2 (extracellular matrix degradation) [40], which may support follicle remodeling in *H. guttatus*. This ovarian-specific enrichment aligns with the species need for efficient lipid utilization during oocyte maturation.

*sox9* showed dramatic gonadal sex bias in *H. guttatus*—140-fold higher expression in testes than ovaries (Figure 5D)—validating its transcriptomic male bias and conserved role as a teleost male differentiation regulator [41,42]. In *Bagridae* (*Pseudobagrus ussuriensis* [7], *Scatophagus argusi* [43]), *sox9* is testis-specific and critical for spermatogenesis, and our data confirm this conservation in *H. guttatus*. Its variable somatic expression reflects additional roles in chondrocyte and neural crest development [44,45], but the gonadal expression reversal (somatic female bias vs. gonadal male bias) underscores a potent testis-specific regulatory program. As a prime target for aquaculture sex control [46], *sox9*’s testis dominance in *H. guttatus* provides a reliable molecular marker for male identification—solving the species’ visual sexing challenge.

*fzd2*, a Wnt signaling receptor [47], showed 10-fold higher expression in *H. guttatus* testes than ovaries (Figure 5E), with low ubiquitous somatic expression. The enrichment of this Wnt pathway component in testes suggests a potential role for Wnt/β-catenin signaling in male gonadal development of *H. guttatus*. This is consistent with the known involvement of Wnt signaling in vertebrate gonad differentiation and patterning [48]. While the specific function of *fzd2* in fish testes requires further study, its marked testis-biased expression identifies it as a candidate regulator in this species.

The qPCR validation confirmed the strong sex-biased expression of these five candidate genes in gonads (Figure 5). Their distinct expression patterns—ranging from strict gonad specificity (*rbm46*) to extreme sex-bias (*sox9*)—highlight their potential as key markers for sex and gonadal state in *H. guttatus*. The functional implications of their expression, such as metabolic support (*myc*), lipid mobilization (*angptl4*), or signaling pathway activity (*fzd2*), provide testable hypotheses for future research into their roles in gonadal differentiation and function.

### 4.3. Biological Pathways from Enrichment Analysis

To elucidate how the DEGs (including the qPCR-validated core genes) synergistically regulate *H. guttatus* gonadal differentiation, we performed KEGG and GO enrichment analyses. These analyses integrate individual gene expression patterns into functional pathways, revealing the systematic regulatory logic underlying sex differentiation.

KEGG enrichment analysis revealed that DEGs are not only involved in sex differentiation and gonadal development but also closely associated with cellular metabolism, structural functions, and gene regulatory mechanisms. The enrichment of TGF-β signaling pathway and GnRH signaling pathway highlights key biological pathways underlying gonadal development and sex determination in *H. guttatus*. These findings provide critical insights into the molecular mechanisms governing sex-related traits in this species.

The TGF-β signaling pathway, a highly conserved system regulating gonadal development across teleosts, was significantly enriched in our dataset. In fish, this pathway is known to influence sex determination and differentiation through multiple mechanisms, including the regulation of steroidogenesis and gonadal cell fate [49,50,51]. Our results showed that 26 DEGs were annotated to this pathway, with 17 forming a cohesive interaction network (Figure 4E). This network included key components such as *acvr2aa, smad6a, bmp5,* and *inhbaa*, suggesting potential involvement in gonadal differentiation processes in *H. guttatus*.

Similarly, the GnRH signaling pathway, which regulates reproductive functions through the hypothalamic-pituitary-gonadal axis, was significantly enriched with 25 annotated DEGs. Among these, 19 genes formed a functional cluster primarily involved in intracellular signal transduction, including members of the phospholipase C family (*plcb2, plcb3, plcb4*) (Figure 4F). This enrichment pattern suggests that neuroendocrine regulation may play an important role in gonadal maturation in *H. guttatus*, consistent with findings in other teleost species where GnRH signaling influences both early gonadal development and reproductive cycles [52,53].

The co-enrichment of the TGF-β and GnRH signaling pathways suggests they may be concurrently active in female gonads. Based on their known functions, these pathways are hypothesized to be involved in processes critical to ovarian function, such as folliculogenesis, a premise that remains to be functionally tested in *H. guttatus*. This observation provides a framework for future functional studies aimed at elucidating the specific mechanisms of sex differentiation in *H. guttatus.* The identification of these significantly enriched pathways, combined with the expression patterns of candidate genes such as *sox9* and *cyp19a1*, offers preliminary insights into the molecular network underlying gonadal differentiation in this species. However, further experimental validation is required to establish the precise functional roles of these pathways in *H. guttatus* sex determination and differentiation.

## 5. Conclusions

In this study, we conducted a comparative transcriptomic analysis of male and female gonads in *H. guttatus*. We identified 3245 DEGs and validated the strong sex-biased expression of five candidate genes (*myc, rbm46, angptl4, sox9*, and *fzd2*) across multiple tissues. Functional enrichment analysis highlighted the significant involvement of the TGF-β and GnRH signaling pathways, suggesting their potential roles in gonadal differentiation and reproductive regulation in this species.

This work provides, to our knowledge, the first comprehensive gonadal transcriptome dataset for H. guttatus, a protected species with limited prior molecular studies. The identified DEGs and enriched pathways establish a foundational resource for investigating its sex determination mechanisms. The extreme sex-biased expression of genes like sox9 offers immediate utility as molecular markers for sex identification, supporting conservation and aquaculture efforts.

We note that the findings, while robust, are based on a correlative transcriptomic approach and a limited sample size. Future studies with larger cohorts and direct functional experiments are needed to validate the precise roles of these candidate genes and pathways in sex differentiation. This study thus provides a crucial molecular framework and defines clear targets for future research aimed at understanding and managing reproduction in *H. guttatus*.

## Figures and Tables

**Table 1:** Primer sequences used for qPCR.

Primer ID	Sequences
EF-1α-F	5′TGCTGCCGTCGCTTTCGT3′
EF-1α-R	5′AAGAGGCTTGTCAGTGGG3′
Myc-F	5′AGAGTTGAAGCAGGAGCAGTT3′
Myc-R	5′GGTTCCTCTGTCTCGTCGTATA3′
Rbm46-F	5′GTGATTGACCGCTCTAAGAACC3′
Rbm46-R	5′GACCGACTGACGATAACTACCA3′
Angptl4-F	5′TTCGGATTGAACAACAGGACAC3′
Angptl4-R	5′GTGGCGTCTCTGGATAATGGT3′
Sox9-F	5′CGCTTGTGCCTATGCCAG3′
Sox9-R	5′AGTCGCCAGAGTTTGCCC3′
Fzd2-F	5′GGAGAGGTGGACTGCGGT3′
Fzd2-R	5′CAGGAAAATGATGGGGCG3′

**Table 2:** Clean Data Results Statistical Table.

Sample	Raw Reads Num	Clean Reads Num	Clean Rate (%)	Q20 (%)	Q30 (%)	GC (%)
C-1	47,877,814	47,844,700	99.93	98.21	93.47	48.43
C-2	56,150,046	56,113,242	99.93	98.12	93.12	49.16
C-3	39,904,316	39,829,582	99.81	96.11	89.83	47.95
X-1	45,576,968	45,525,206	99.89	96.75	91.70	45.99
X-2	49,653,288	49,613,862	99.92	98.28	93.81	46.08
X-3	55,643,880	55,606,622	99.93	98.27	93.67	48.34

**Table 3:** Statistics on the comparison of samples with the reference genome.

Sample	Total Read Pairs	Total Mapped Reads	Uniq Mapped Reads	Multiple Mapped Reads	Postive Map	Negative Map
C-1	23,922,350	22,798,746	21,178,933	1,619,813	21,178,933	21,178,933
C-2	28,056,621	26,909,450	24,398,926	2,510,524	24,398,926	24,398,926
C-3	19,914,791	17,942,240	16,749,531	1,192,709	16,749,531	16,749,531
X-1	22,762,603	20,514,260	19,517,985	996,275	19,517,985	19,517,985
X-2	24,806,931	23,359,339	21,896,799	1,462,540	21,896,799	21,896,799
X-3	27,803,311	26,457,697	24,402,001	2,055,696	24,402,001	24,402,001

**Table 4:** Statistics on The Number of Genes in Different Expression Level.

Sample	Expressed_Gene	Total _Gene	0	0~1	1~3	3~15	15~60	>60
C-1	19,429	34,404	14,975 (43.53%)	6518 (18.95%)	2399 (6.97%)	4151 (12.07%)	3653 (10.62%)	2708 (7.87%)
C-2	18,717	34,404	15,687 (45.60%)	6801 (19.77%)	2278 (6.62%)	3917 (11.39%)	3295 (9.58%)	2426 (7.05%)
C-3	18,703	34,404	15,701 (45.64%)	6031 (17.53%)	2118 (6.16%)	3794 (11.03%)	3847 (11.18%)	2913 (8.47%)
X-1	23,157	34,404	11,247 (32.69%)	5256 (15.28%)	2804 (8.15%)	6185 (17.98%)	6214 (18.06%)	2698 (7.84%)
X-2	22,903	34,404	11,501 (33.34%)	5357 (15.57%)	2559 (7.44%)	6431 (18.69%)	6028 (17.52%)	2528 (7.35%)
X-3	19,227	34,404	15,177 (44.11%)	6807 (19.79%)	2069 (6.01%)	3896 (11.32%)	3706 (10.77%)	2749 (7.99%)

## Data Availability

The data from the study can be searched in Entrez using PRJNA1291962.
